# Characterization of bacterial diversity and screening of cellulose-degrading bacteria in the gut system of *Glenea cantor* (Fabricius) larvae

**DOI:** 10.3389/fbioe.2024.1340168

**Published:** 2024-02-22

**Authors:** Ran-Ran Su, Bi-Qiong Pan, You-Xi Luo, Xia-Lin Zheng, Wen Lu, Xiao-Yun Wang

**Affiliations:** Guangxi Key Laboratory of Agric-Environment and Agric-Products Safety, National Demonstration Center for Experimental Plant Science Education, College of Agriculture, Guangxi University, Nanning, China

**Keywords:** gut bacterial community, *Glenea cantor*, illumina MiSeq 16S rRNA sequencing, lignocellulose degradation, cellulose degrading bacteria

## Abstract

The intestinal bacteria of longhorn beetles would be ideal targets for pest control and lignocellulosic resources by destroying or exploiting their cellulose-degrading function. This article aims to investigate the diversity and community structure of intestinal bacteria the oligophagous longhorn beetle *Glenea cantor*. Additionally, it seeks to identify the presence of lignocellulose-degrading bacteria in the gut, and explore their role in consuming host kapok trees *Bombax malabaricum*. In this study, the bacterial community from *G. cantor* was examined by Illumina sequencing of 16S ribosomal RNA (rRNA) targeting the V3 and V4 regions. A total of 563,201 valid sequences and 814 OTUs were obtained. The dominant phyla were Proteobacteria, and the dominant genera were *Acinetobacter* and Lactococcus. The analysis of microbial diversity revealed a high bacterial diversity in the samples, with the gut bacteria playing a crucial role in the physiological activities of the host, particularly, 9 genera of intestinal bacteria with cellulose degradation function were found, highlighting their vital role in cellulose degradation. Five strains of cellulose-degrading bacteria, belonging to the genus *Pseudomonas,* were obtained from the intestinal tract of *G. cantor* larvae using traditional isolation and culture techniques as well as 16S rDNA sequencing. Among these strains, A4 exhibited a cellulase activity of 94.42 ± 0.42 U/mL, while A5 displayed the highest filter paper enzyme activity of 127.46 ± 3.54 U/mL. These results offered valuable insights into potential targets for pest control through internal attack digestion and cellulose-degrading bacteria in longhorn beetles.

## 1 Introduction

*Glenea cantor* Fabricius (Coleoptera: Cerambycidae: Lamiinae) is widely distributed in Vietnam and southern China (Lu et al., 2007). Under suitable conditions, it can reproduce round without dormancy and diapause all year (Lu et al., 2011). *Glenea cantor* is oligophagous and can cause serious damage to *Bombax malabaricum*, an important landscaping tree species (Wu et al., 2022). The females laid eggs in slots on the branches with weak growth vigor (Lai et al., 2008). The methods for controlling these beetles mainly include manual trapping and killing of adults, timely removal of dead branches and debris to reduce the insect population and use chemical control (Zhong et al., 2011). Due to its extensive egg production, high hatching rate and strong reproductive ability (Lai et al., 2008; Lu et al., 2013; Dong et al., 2017), it is an ideal model for the study of management and efficient cellulose degradation in longhorn beetles. As an oligophagous pest, the integrated measurement of *G. cantor* could be developed by breaching its internal digestion system to achieve (Tokuda, 2019). As a member of Cerambycidae, the wood degradation mechanism of *G. cantor* would also contribute to research on other devastating longhorn beetles, such as *Monochamus alternatus* and *M. galloprovincialis*, the vectors of *Bursaphelenchus xylophilus*, a pine wood nematode that causes pine wilt disease (Alves et al., 2016; Guo et al., 2020).

The cellulose degradation of longhorn beetles is related to their structure of host plants and intestinal cellulase and hemicellulase activities (Luo et al., 2019). The structure of longhorn beetles’ host plant wood is composed of varying amounts of lignin, cellulose and hemicellulose arranged in a certain pattern (Pauchet et al., 2014). Longhorn beetles inhabit wood and can digest cellulose and hemicellulose, which are the primary constituents of wood (Li et al., 2020a). The wood consumption habits and characteristics of the longhorn beetle derive from digestive enzymes, which may be from endogenous enzymes or/and intestinal flora (Willis et al., 2010; Guan et al., 2011; Li et al., 2020c). Firstly, several endogenous cellulase genes had been cloned from different beetles, including the yellow-spotted longhorn beetle *Psacothea hilaris* (Sugimura et al., 2003), the mulberry longhorn beetle *Apriona germari*, *Bateocera horsfieldi* (Wei et al., 2006a; Wei et al., 2006b; Xia et al., 2013), *M. alternatus* (Li et al., 2020b) and *Mesosa myops* (Liu et al., 2015). Secondly, the symbiotic microorganisms of insects, such as bacteria and fungi, are traditionally believed to produce cellulase and other digestive enzymes for nutrients (Shelomi and Chen, 2020; Li et al., 2021). Insect growth and development were affected by microorganisms that directly or indirectly participate in their metabolism (Ayayee et al., 2016). To some extent, the intestinal microbial function of insects were determined by the structure of their intestinal microbial community (Hooper and Gordon, 2001). As a result, the study of gut microbiota has emerged as a novel field concerning the ecological and functional dynamics of the intestinal microbial habitats (Jang and Kikuchi, 2020).

The methods of gut microbiota research include the culture-based method, culture-independent method, and Amplicon-based taxonomic identification, and so on (Romero et al., 2019). A range of bacteria from the insect gut have been identified using traditional isolation and culture techniques (O'Sullivan, 2000). However, culture-dependent bacterial isolation methods may introduce bias in the characterization of microbial communities, as not all bacteria can be cultured in the laboratory (Brock, 1987). 16S rRNA technology has become a widely recognized method which can reflect the dynamic changes of intestinal microbial ecological community structure, and it provides a more accurate means of detecting and identifying both known and unknown bacteria in intestinal flora (Munoz-Benavent et al., 2021). This technology overcomes the complex problems of traditional methods such as morphological examination, isolation and culture, and has sufficient variation to differentiate between most bacterial species (Nguyen et al., 2016). By constructing a library based on 16S rRNA sequencing to analyze the structure and function of intestinal bacteria, we learned that bacteria occupy a dominant position in the intestinal microbiota of beetles, and the dominant flora is relatively stable. For instance, the dominant phyla of *Stromatium barbatum* were Actinomycetes, Proteobacteria, and Firmicutes (Yadav et al., 2022). Similarly, *Cacosceles newmannii* exhibited dominance of Proteobacteria (Javal et al., 2023), while *Monochamus saltuarius* mainly harbored Proteobacteria and Firmicutes (Ge et al., 2021). Additionally, the main intestinal microorganisms of *Anoplophora glabripennis* consisted of the phyla Proteobacteria, Actinobacteria, Bacteroidetes, and Firmicutes (Schloss et al., 2006). Thus, the dominant bacterial phyla in the intestinal bacteria of longhorn beetles consist primarily of Proteobacteria, Firmicutes, Actinobacteria, and Bacteroidetes. Although 16S rRNA gene analysis is a valuable approach to study difficult-to-cultivate microorganisms, it overcomes the limitations of traditional isolation methods. It can effectively detect and identify both known and unknown bacteria in the intestinal flora (Munoz-Benavent et al., 2021). However, it is not without its challenges. The relatively accuracy is low in identifying bacterial species. Additionally, the dominant populations may mask the presence of rare microbial communities. To obtain a more comprehensive understanding of insect gut microbiota, long-term studies have demonstrated that a combination of culture-dependent and high-throughput sequencing technologies is necessary (Wang et al., 2020).

The larvae of longhorn beetles exhibit a remarkable capacity for cellulose and lignin degradation, with their intestinal microorganisms playing a vital role in facilitating this process. Therefore, extensive research had been conducted on the screening and functional analysis of cellulose-degrading bacteria in longhorn beetles. The types of intestinal cellulose-degrading bacteria varied depending on the species of longhorn beetles, their hosts and habitats. For instance, the intestinal tract of *Batocera lineaolata* larvae contained strains belonging to the genus *Ochrobactrum* and *Raoultella*, which exhibited high cellulase production (Yang et al., 2021). Similarly, *Bacillus subtilis*, derived from the gut of *Aromia bungii* larvae, with high cellulase activity, had the ability to enhance the protein content of broiler chickens and improve their intestinal microflora (Su et al., 2015). The cellulose-degrading bacteria derived from the pre-midgut of *Apriona germari* larvae belong to the genus *Cellulamonas* and *Bacillus subtilis* (He et al., 2001; Xie et al., 2020). Longhorn beetles are a diverse group of insects, with approximately 35,000 known species worldwide. In China, more than 2,000 species have been reported. These beetles mainly feed on woody plants (Jin et al., 2019). They develop intestinal microorganisms that acquired the ability to break down cellulose and lignin, giving them a competitive advantage with long-term domestication of host plants (Dar et al., 2022; Yadav et al., 2022; Xie et al., 2023). Despite the abundance of longhorn beetles, there is insufficient research on their cellulose degradation mechanisms.

The ability of longhorn beetles to degrade cellulose primarily depends on cellulase. Cellulase production was carried out by symbiotic bacteria in the gut, such as *Batocera lineaolata* (Yang et al., 2021), *Aromia bungii* (Su et al., 2015), and *Apriona germari* (Xie et al., 2020). Additionally, longhorn beetles themselves produce proteins encoded by their own cellulase genes, for example, *Batocera horsfieldi* (Mei et al., 2016), *Monochamus alternatus* (Li et al., 2020c), and *Anoplophora malasiaca* (Chang et al., 2012). In some cases, they may produce cellulase using both mechanisms, for instance, *Apriona germari* (Wei et al., 2006a; Wei et al., 2006b; Xie et al., 2020). Cellulase with endoglucanase activity, cellobiase activity, and filter paper enzyme activity mainly existed in midgut of *G. cantor* (Yang et al., 2011). However, it was unknown that the source of its enzyme production originated from intestinal microorganisms, the beetles themselves, or both. From this perspective, the comparative 16S rRNA sequencing method was used to obtain gut bacterial diversity analysis of the larval gut and frass directly. Cellulose-degrading bacteria were screened and identified by traditional isolation culture and 16S rDNA bacterial identification technology, which derived from the gut of *G. cantor*. These results would contribute to the bacterial community structure and diversity, the function of the intestinal bacteria, the digestive mechanism and the possible gut bacteria targets for developing novel control method in *G. cantor*.

The complexity of cellulose structure (Tsegaye et al., 2019) and the low activity of cellulose-degrading enzymes in nature pose challenges in efficiently degrading and utilizing cellulose resources in practical applications (Zhu and Pan, 2022). Consequently, the high-value utilization of cellulose as a resource is limited. This study aims to analyze the structure and function of the intestinal microbial community and screen target strains in *G. cantor* larvae. The findings provide a foundation for understanding the mechanism of insect cellulose degradation and offer valuable insights for utilizing insect intestinal microorganisms in cellulose degradation, based on the cellulose-degrading properties of insect-derived cellulases.

## 2 Materials and methods

### 2.1 Insect rearing

*Glenea cantor* was collected from kapok trees in Qingxiu Mountain (22°12′∼23°32′N, 107°45′∼108°51′E), Nanning, China. When an adult came out of the wood, the male and female adults were placed in a glass bottle for mating, and a small section of coarse kapok, about 4 cm in diameter and 6 cm in length, was provided as food. The cuticle with the eggs were cut out with a knife and cultured in a petri dish containing moist cotton. The eggs were incubated until the larvae reached the fourth instar. *G. cantor* were reared under controlled conditions with an indoor temperature of 25°C ± 1°C, a relative humidity of 70% ± 5% and a photoperiod of 14L: 10D.

### 2.2 Analysis of bacterial diversity in the intestinal tract of *G. cantor* larvae

#### 2.2.1 Gut dissection and frass sample collection

The fourth instar larvae of *G. cantor* with similar body weight were randomly divided into three groups. Fresh frass samples from larvae were collected daily at 7 P.M. for a duration of 6 days. These frass samples were then stored at −20°C before DNA extraction. The larval frass samples were labeled as GcLF. The detailed information of insects and frass samples was listed in Supplementary Table S1 for reference. Five larvae were grouped together as a repetition and transferred to a moist, sterile culture dish. They were starved for 48 h to empty the intestinal food residue. The larvae were washed with sterile water and subsequently treated with 75% alcohol for surface sterilization, then rinsed with sterile water, repeated the process three times. Under the sterile conditions, the larvae of *G. cantor* were dissected by removing the head and cutting along the back line of the cuticle in PBS solution. The intestines were then extracted from the body using tweezers. The surface of the intestines was carefully stripped of any irrelevant material. The extracted intestines were placed in a 1.5 mL centrifuge tube containing PBS buffer and frozen in liquid nitrogen. Finally, the samples were stored at −80°C. The larval gut of *G. cantor* was labeled as GcLG.

#### 2.2.2 Microbial DNA extraction and PCR amplification

DNA samples were extracted using the HiPure Tissue DNA Kits and HiPure Stool DNA Kits (Magen, Guangzhou, China) following the manufacturer’s protocols. The concentration of DNA was assessed by Nanodrop 2000C (Thermo Scientific, Waltham, MA, USA), and purity was monitored by 1% agarose gel electrophoresis. DNA was diluted to a concentration of 100 ng/μL with sterile water. For PCR amplification, the V3-V4 region of the bacterial 16S rRNA gene was performed using the primers (341F: CCTACGGGNGGCWGCAG; 806R: GGACTACHVGGGTATC TAAT) (Guo et al., 2017). PCR reactions were performed in triplicate using a 50 μL mixture containing 5 μL of 10 × KOD Buffer, 5 μL of 2 mM dNTPs, 3 μL of 25 mM MgSO_4_, 1.5 μL of each primer (10 μM), 1 μL of KOD Polymerase, and 100 ng of template DNA.

#### 2.2.3 Processing of sequence data

Amplicons were extracted from 2% agarose gels, purified using the AxyPrep DNA Gel Extraction Kit (Axygen Biosciences, Union City, CA, U.S.) and quantified by ABI StepOnePlus Real-Time PCR System (Life Technologies, Foster City, USA). The purified amplicons were pooled in equimolar and paired-end sequencing (PE250) on an Illumina platform according to the standard protocols. FASTP was used to excluded low-quality adapters and reads which contained more than 10% of unknown nucleotides and less than 50% of bases with quality (Q-value) > 20 (Chen et al., 2018). Then the achieved paired end clean reads were merged as raw tags using FLSAH (version 1.2.11) with a minimum overlap of 10 bp and a mismatch error rate of 2% (Magoč and Salzberg, 2011). Raw tags were filtered by QIIME (version 1.9.1) pipeline to obtain the high-quality clean tags (Caporaso et al., 2010). Finally, the effective tags were obtained by removing all chimeric tags which were found by reference-based chimera checking via UCHIME algorithm by searching against the reference database (version r20110519, http://drive5.com/uchime/uchime_download.html, accessed in July 2020) (Edgar et al., 2011).

#### 2.2.4 Data analysis

The effective tags were clustered into operational taxonomic units (OTUs) of  ≥ 97% similarity using UPARSE (version 9.2.64) (Edgar, 2013). Between groups Venn analysis of achieved OTUs was performed in the R project Venn Diagram package (version 1.6.16) (Chen and Boutros, 2011). The tag sequence with highest abundance was selected as representative sequence within each cluster and were classified into organisms by a naive Bayesian model with the RDP classifier (version 2.2) based on Greengene database (version gg_13_5) (DeSantis et al., 2006; Wang et al., 2007). The stacked bar plot of the community composition was visualized in R project ggplot2 package (version 2.2.1) (Wickham and Chang, 2008). Chao1, Simpson and all other alpha diversity index were also calculated in QIIME (Caporaso et al., 2010). The comparison of alpha index between groups was calculated by Welch’s *t*-test and Wilcoxon rank test in the R project Vegan package (version 2.5.3) (Dixon, 2003). The KEGG pathway analysis of the OTUs was inferred using Tax4Fun (version 1.0) and PICRUSt2 (version 2.1.4) (Langille et al., 2013; Aßhauer et al., 2015).

### 2.3 Isolation and identification of cellulose-degrading bacteria in the intestinal tract of *G. cantor* larvae

#### 2.3.1 Homogenate preparation from the intestinal tract of *G. cantor* larvae

**FIGURE 1:**
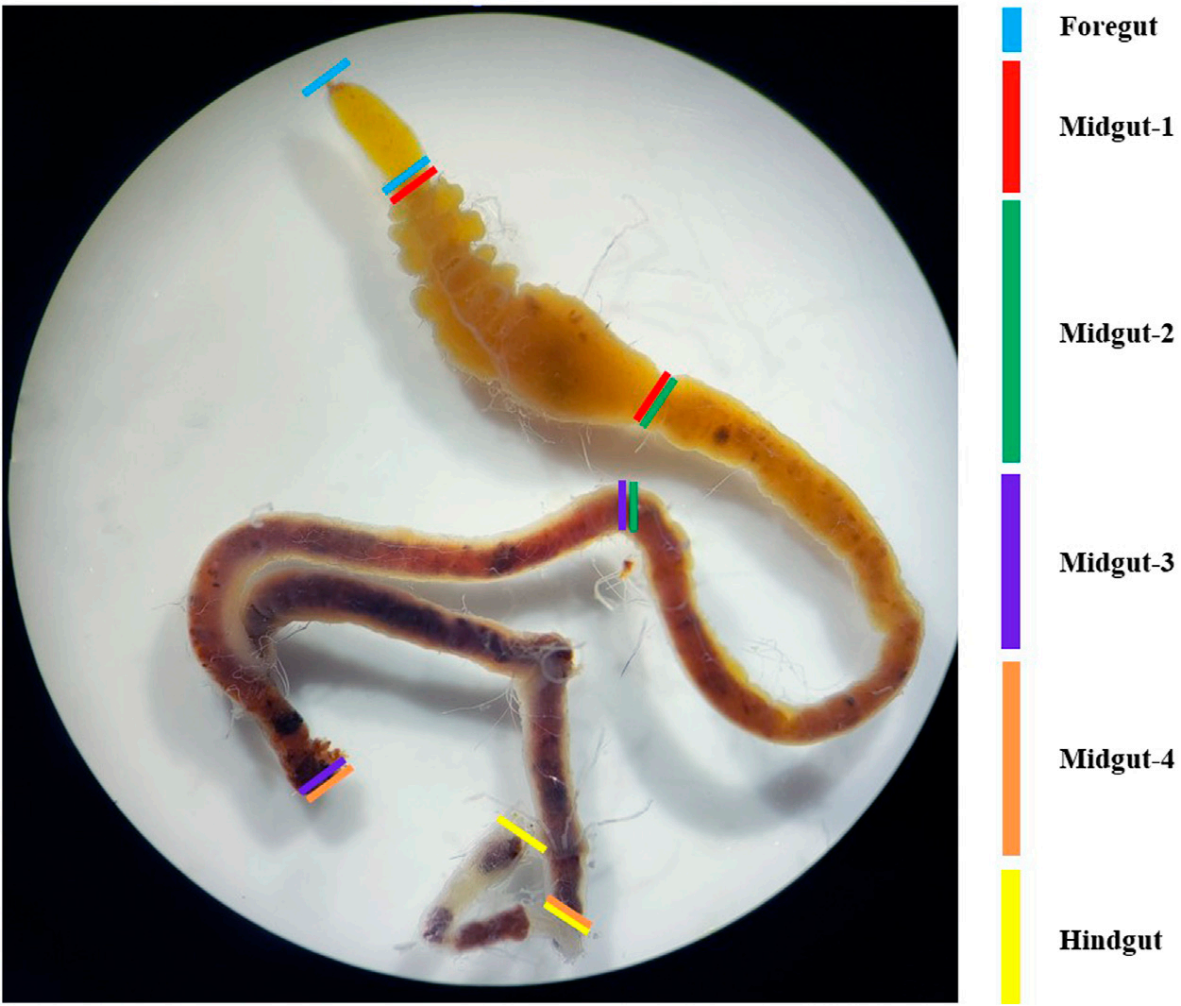
Sectional morphology of the intestinal tract of the fourth instar of *G. cantor*. Note: To determine the pH of each tissue in the intestinal tract of *G. cantor*, fourth-instar larvae were collected and dissected to obtain the complete intestinal tract on a dissection plate. The intestinal tract contents were squeezed out on a wide range of pH test paper, and the preliminary pH were estimated by comparing the color of the test paper with the color chart. For accurate measurement, precision pH test paper was used based on the preliminary results (Supplementary Table S2).

The intestinal tract of the fourth-instar larvae of *G. cantor* were close to neutral, and pH 7 should be selected when screening intestinal bacteria based on our pH measurement of different intestinal sections (Figure 1; Supplementary Table S2). Ten fourth-instar larvae with a similar size were selected and subjected to a 48-hour starvation period to remove any remaining food residues from their intestines. The dissection process was carried out under the super clean workbench after 30 min of UV treatment. Initially, the larvae were cleansed by immersing them in sterile water for 30 s, followed by a 1-minute soak in 75% alcohol to sterilize their body surfaces. This was followed by another 10-second rinse in sterile water. The process was repeated three times. During the dissection, the epidermis of the larvae was carefully cut along the top line by the sterilized scissors. The intestine was then gently extracted using tweezers, while ensuring the removal of any fat or other substances attached to its surface. The obtained intestinal tract was placed in a 2.0 mL centrifuge tube containing 500 μL of sterile water. It was thoroughly ground using a grinding pestle, and sterile water was added to achieve a final volume of 1 mL. Subsequently, 50% glycerol was added, and the resulting solution was mixed with the intestinal bacteria solution in a 1:1 ratio. This mixture was then stored in a −80°C refrigerator as the stock solution of longhorn beetles’ larval intestines for future use.

#### 2.3.2 Enrichment culture and primary screening of cellulose-degrading bacteria

For enrichment purposes, 1 mL of each gut homogenate was separately inoculated into conical flasks containing 100 mL of the enrichment medium (All components of the culture medium were listed in Supplementary Table S3). The inoculated culture media were incubated at 37°C and 200 r/min for 48 h. The cultured bacterial solution was serially diluted in sterilized water from 10^−3^ to 10^−8^ dilution. Then, 100 μL of each dilution was spread in three duplicates on the Congo red cellulose Sodium medium plates. The sterile water control group 1 and empty medium control group 2 were added. The plates were incubated under aerobic conditions at 37°C for 48 h in case of cellulose-degrading bacteria. The cellulose degradation circles were observed in the plates. Next, the bacterial colonies, which produced the cellulose degradation circles, were picked and transferred to LB solid medium plates for purification. The isolated bacteria were purified through the repeated streaking methods, codified and preserved for further examination. The purified single colonies were incubated overnight at 37°C and 200 r/min. Finally, they were mixed with 50% glycerol in a 1:1 ratio and stored it at −80 °C for future use.

The preserved strains were diluted to a concentration of 10^−5^ and spread on a medium containing Congo red cellulose sodium. Four sets of biological replicates were prepared for each strain and cultured upside down at 37°C for 48 h. Subsequently, colonies from each group were selected from the solid medium of sodium carboxymethyl cellulose. From each plate, 3 colonies were picked and a total of 5 plates were used in each group. The selected strains were then cultured at 37°C for 48 h. To evaluate the hydrolysis zone, the strains on the petri dish were first stained with a 1% Congo red staining solution for 30 min, followed by decolorization with a 1 mol/L NaCl solution for another 30 min. After decolorization, the colony diameter of the hydrolysis zone was measured and recorded using an electronic vernier caliper. The ability of cellulose-degrading bacteria to degrade cellulose can be compared by the ratio of the diameter of the transparent circle (D) to the diameter of the colony (d). The larger the ratio of D/d, the stronger the ability of the corresponding strain to degrade cellulose, and *vice versa*.

#### 2.3.3 Rescreening of cellulose-degrading bacteria

##### 2.3.3.1 Crude enzyme solution extraction

The strains that produced the cellulose degradation circles should be inoculated into the enzyme-producing medium with a 1% inoculum. The cultivation should be carried out for 48 h at a temperature of 37°C and a speed of 180 r/min. After cultivation, the mixture should be centrifuged at 5000 r/min at 4°C for 15 min. The resulting supernatant is the crude enzyme solution, which should be stored at 4°C for future use.

##### 2.3.3.2 Filter paper enzyme activity (FPA) and cellulase activity (CMCA)

The Filter Paper Activity (FPA) was measured using the DNSA method (Miller, 1959). The strips of qualitative filter paper measuring 1 cm × 6 cm were cut and placed at the bottom of a test tube. Next, 1.5 mL of Hac-NaAc buffer solution (pH = 4.8) and 1 mL of crude enzyme solution were added. The mixture was then incubated in a constant temperature water bath at 50°C for 30 min. After that, 2.5 mL of DNS reagent was added and the solution was boiled for 5 min. Subsequently, the mixture was diluted to 10 mL using distilled water and the OD value was measured at a wavelength of 540 nm. The enzyme activity unit (IU) was defined as follows: 1 mL of crude enzyme solution produced 1 μg of reducing sugar within 1 min.

Cellulase activity was measured using the DNSA method (Miller, 1959). To perform the measurement, 1 mL of prepared crude enzyme solution was added to a test tube. Then, 1.5 mL of Hac-NaAc buffer solution with 1% CMC-Na was added to the same test tube. The mixture was thoroughly mixed and kept at 50°C for 30 min. After that, DNS solution (2.5 mL) was added to each test tube and shaken well. The test tubes were then placed in a boiling water bath and boiled to stop the enzymatic reaction for 5 min. The absorbance value was measured at a wavelength of 540 nm after quickly cooling down the mixture and adjusting the volume to 10 mL. The obtained value was substituted into the glucose calibration curve to calculate the corresponding value A.

The enzyme activity of FPA/CMCA was calculated using the following formula:

$$\text{FPA}/\text{CMCA }\left(U/\text{mL}\right)=\frac{A\times n\times 1000}{t\times v}$$

Note: The value A is obtained by substituting the measured absorbance value into the standard curve equation (Supplementary Figure S1, see the attachment for standard curve preparation and Supplementary Table S4). Here, n represents the dilution ratio of the solution, 1000 is the conversion factor from mg to μg, t represents the color development time, and v represents the volume of the crude enzyme solution.

#### 2.3.4 Identification and phylogenetic analysis of cellulose-degrading bacteria

The morphological identification and physiological characteristics of cellulose-degrading bacteria can be determined by observing and recording the color, shape, and texture of a single colony under an optical microscope after plate culture. The identification of bacterial cell morphology follows the methods outlined in the “Bergey’s Bacteria Identification Manual,” which includes identifying bacterial colony color, bacterium shape, size, and distinguishing between Gram-negative and Gram-positive bacteria. Molecular identification of cellulose-degrading bacteria involves extracting the total DNA of each bacteria using a bacterial DNA extraction kit. Universal bacterial primers 27F (AGTTTGATCMTGGCTCAG) and 1492R (GGT​TAC​CTT​GTT​ACG​ACT​T) are then used to amplify bacterial 16S rDNA. Subsequently, 16S rDNA sequencing is performed to construct a phylogenetic tree and perform cluster analysis to identify the species of cellulose-degrading bacteria. The 16S rDNA gene sequence of the cellulose-degrading bacteria strains found in the larval gut of *G. cantor* was compared with known 16S rDNA sequences in the NCBI database (https://www.ncbi.nlm.nih.gov/) using BLAST. The highly similar and reliable sequences were downloaded for clustering analysis. The obtained sequences and target gene sequences were analyzed by MEGA 11.0 to construct a Neighbor-joining phylogenetic tree. The bootstrap analysis was set to 1000 repetitions to calculate the support rate of each branch.

## 3 Results

### 3.1 Gut bacterial community of *G. cantor* larvae

#### 3.1.1 Intestinal and fecal bacterial diversity of *G. cantor* larvae

**FIGURE 2:**
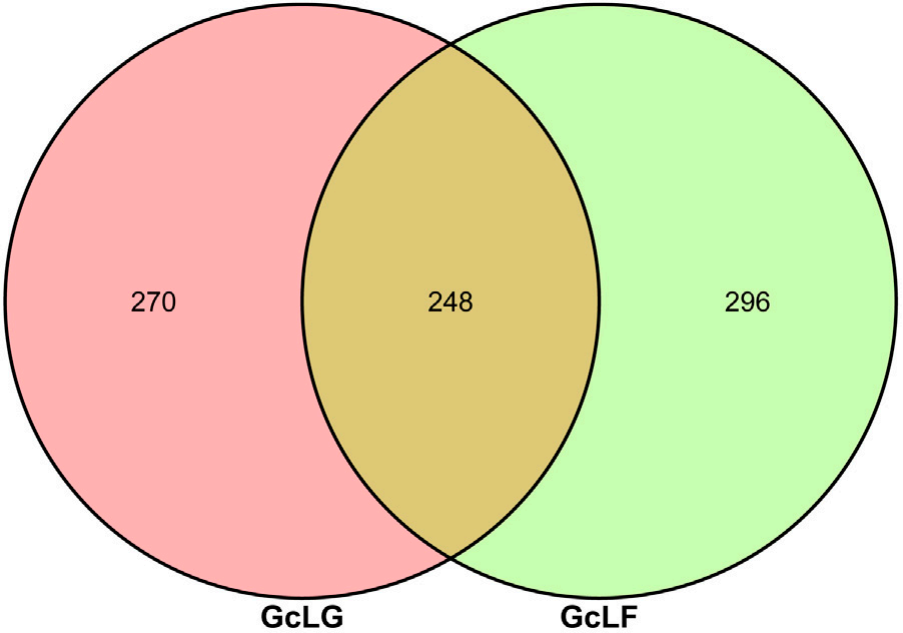
Veen diagram of OTUs of bacteria in intestinal and frass samples from the larva of *G. cantor*. Note: GcLG: intestinal sample; GcLF: frass sample.

Through Illumina HiSeq of 16S rRNA gene amplicon sequencing, 643, 626 raw pair end reads were obtained from 6 samples (Supplementary Data Sheet S1). After quality filtering and chimera removal, 563,201 (87.5%) effective tags remained for analysis. These tags were clustered into 814 OTUs at 97% sequence identity. The average value of common and unique OTUs between the two groups of GcLG and GcLF were analyzed and viewed by a Venn diagram (Figure 2). There were 248 OTUs shared between each component in the group, which accounted for 47.88% and 45.59% of the total number of OTUs in each sample, respectively. Additionally, there were 270 and 296 unique OTUs within each group, accounting for 52.12% and 54.41% of the total number of OTUs in the sample.

Bacterial alpha diversity indexes, including ace, chao, Simpson, Shannon and sobs were compared between larval gut and frass samples (GcLG and GcLF) in *G. cantor* (Table 1). The species abundance and diversity of GcLF were higher than that of GcLG, but the difference between GcLG and GcLF were not significant, which indicated that the dominant bacteria of larval gut may be transferred to the feces through the digestive tract. Therefore, the analysis of fresh fecal bacteria may reflect larval intestinal status.

**TABLE 1:** Bacterial alpha diversity in larval gut and frass samples (GcLG and GcLF) in *G. cantor*.

Index	GcLG	GcLF	T test *p*-value	Wilcoxon *p*-value
ace	563.16 ± 14.14	643.12 ± 52.53	0.11	0.1
chao	553.02 ± 24.41	620.03 ± 39.02	0.08	0.2
Simpson	0.90 ± 0.05	0.92 ± 0.02	0.63	0.7
Shannon	4.72 ± 0.82	4.97 ± 0.43	0.67	0.7
sobs	460.00 ± 5.57	522.33 ± 60.75	0.22	0.2

**FIGURE 3:**
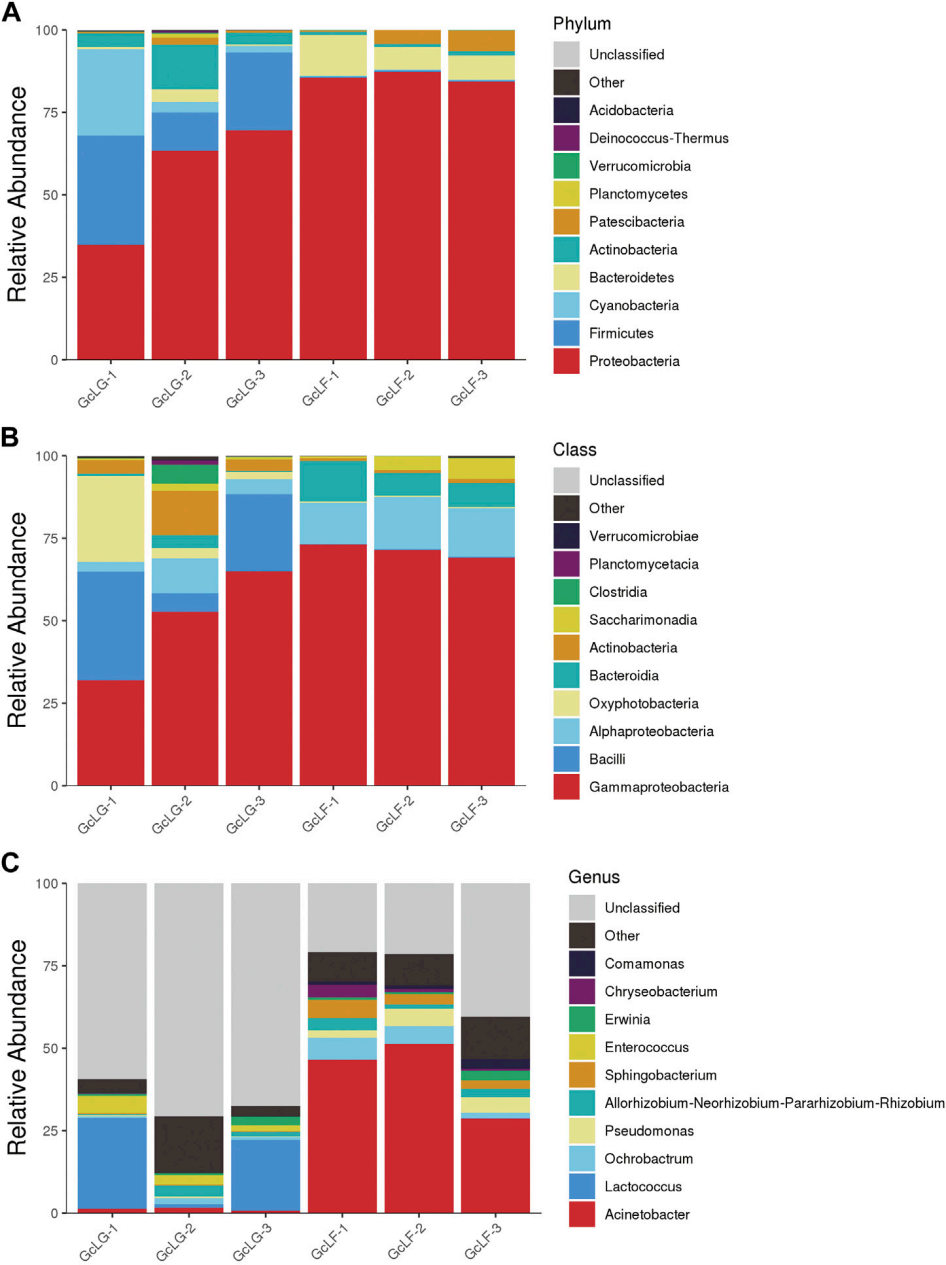
Bacterial community composition in larval gut and frass samples in *G. cantor*. Note: **(A)** Bacterial community composition at phylum level; **(B)** Bacterial community composition at class level; **(C)** Bacterial community composition at genus level. Only the relative abundance of top 10 species were listed at relative levels. “Other” indicates the relative abundance of species that not included but could be identified at relative level; thus “unclassified” indicates species that could not be identified.

Bacterial community composition were analyzed at phylum, class and genus level which only the relative abundance of top 10 species was listed in larval gut and frass samples (GcLG and GcLF) of *G. cantor* (Figure 3). The six most abundant phyla were Proteobacteria, Firmicutes, Cyanobacteria, Bacteroidetes, Actinobacteria and Patescibacteria in gut samples. The first four phyla accounted for 96% of all the reads. Similarly, the four most abundant phyla of frass samples were Proteobacteria, Bacteroidetes, Patescibacteria, and Actinobacteria, which account for 99% of all the reads. Overall, Proteobacteria was the most common phylum, accounting for an average of 71% of all the reads (Figure 3A). At class level, Gammaproteobacteria was the most abundant with 50% and 71% of reads in larval gut and frass samples (Figure 3B). At genus level, *Lactococcus* and *Enterococcus* were the two most abundant of larval gut samples, accounting for 17% and 3% of all reads. In contrast, *Acinetobacter* and *Ochrobactrum* were the two most abundant of larval frass samples, making up 42% and 5% of all reads (Figure 3C).

#### 3.1.2 Function analysis with aspects to wood digestion

**FIGURE 4:**
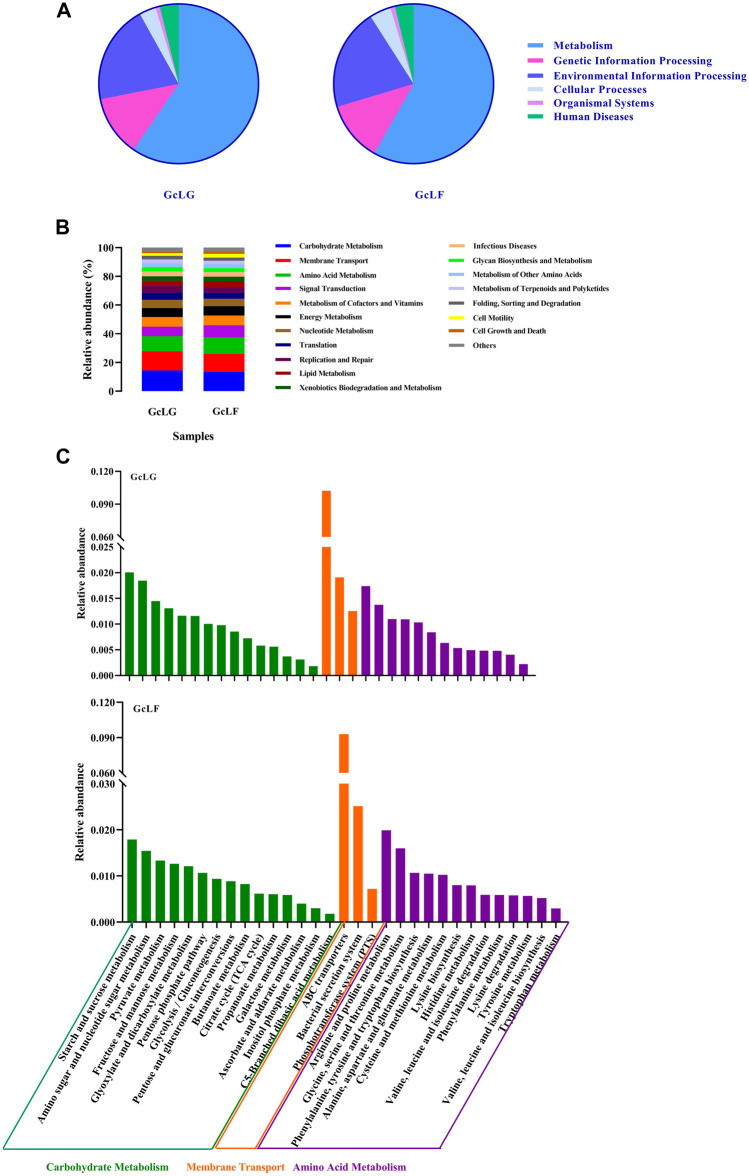
The KEGG function analysis of intestinal and fecal in *G. cantor* larvae. Note: GcLG: intestinal sample; GcLF: frass sample. **(A)** The pie chart of KEGG function analysis based on Tax4Fun algorithms. **(B)** The stacked diagram of KEGG functional analysis based on PICRUST2 algorithms. The diagram is only for metabolic pathways whose relative abundance was greater than 1%. **(C)** The results of KEGG functional analysis in Carbohydrate Metabolism, Membrane Transport and Amino Acid Metabolism based on Tax4Fun algorithms.

The distribution of functional genes in the metabolic pathways of the gut and larval fecal microbial communities of *G. cantor* were shown in Figure 4. The bacterial flora of the gut and feces primarily contributed to metabolic activities, genetic information processing, and environmental information processing (Figure 4A). Upon averaging, the functions can be ranked from high to low according to the relative abundance ratio (greater than 1%): Carbohydrate Metabolism (14.02%), Membrane Transport (12.96%), Amino Acid Metabolism (10.94%), Signal Transduction (7.54%), Metabolism of Cofactors and Vitamins (6.72%), Energy Metabolism (6.41%), Nucleotide Metabolism (5.41%), Translation (4.60%), Replication and Repair (4.34%), Lipid Metabolism (3.61%), Xenobiotics Biodegradation and Metabolism (3.36%), Infectious Diseases (3.29%), Glycan Biosynthesis and Metabolism (2.83%), Enzyme Families (2.76%), Metabolism of Other Amino Acids (2.55%), Folding, Sorting and Degradation (2.32%), Cell Motility (2.25%), Cell Growth and Death (1.37%) (Figure 4B). The results indicated that the intestinal bacterial flora of *G. cantor* was mainly involved in carbohydrate metabolism, membrane transport, and amino acid metabolism, as well as partial lipid, nucleotide metabolism and biodegradation of the host. In carbohydrate metabolism, the bacterial flora primarily participated in starch and sucrose metabolism, amino sugar and nucleotide sugar metabolism, and pyruvate metabolism. Additionally, they played a role in arginine and proline metabolism, glycine, serine and threonine metabolism, and phenylalanine, tyrosine and tryptophan biosynthesis in Amino Acid Metabolism. Furthermore, the bacterial flora was involved in the membrane transport, particularly ABC transporters and bacterial secretion system (Figure 4C). There was no significant difference in the abundance of these metabolic functions between the larval intestine and larval frass. The biodegradation function indicated that intestinal bacterial flora participated in the degradation of certain substances (such as starch and sucrose, amino sugar, nucleotide sugar and amino acid) of the host in these metabolic pathways.

Based on the results of OTUs’ annotation of species, the lignocellulose-degrading bacteria were found at the genus level, as shown in Table 2. Cellulose-degrading bacteria were found at the genus level belonging to 12 genera. Among them, the abundance information ratio of cellulose-degrading bacteria belonging to 4 genera, namely *Microbacterium*, *Streptomyces*, *Lachnospiraceae_NK4A136_group* and *Gordonia,* were higher in the gut compared to fecal samples. On the other hand, the abundance information of 8 genera of cellulose-degrading bacteria, including *Chryseobacterium*, *Stenotrophomonas*, *Flavobacterium*, *Cellvibrio*, *Acinetobacter*, *Paenibacillu*s, *Pseudomonas* and *Sphingobacterium*, were lower than that in fecal samples. Notably, the abundance of *Chryseobacterium*, *Acinetobacter*, and *Stenotrophomonas* in fecal samples was much greater than that in intestinal samples, and their GcLF/GcLG values were 41.69, 34.72, and 22.51, respectively.

**TABLE 2:** At the genus level, bacterial with lignocellulose decomposition ability obtained in larval gut and frass samples (GcLG and GcLF) in *G. cantor*.

Genus	Tag abundance information		Fold (GcLF/GcLG)	*p*-value	Function	References
	GcLG	GcLF				
*Chryseobacterium*	0.04	1.80	41.69	0.23	cellulose degradation	Kognou et al. (2022)
*Stenotrophomonas*	0.07	1.55	22.51	0.13	cellulose degradation	
*Flavobacterium*	0.10	1.47	14.19	0.00	cellulose degradation	Kim and Yu (2020)
*Microbacterium*	0.07	0.02	0.31	0.43	cellulose degradation	Soares et al. (2012)
*Cellvibrio*	0.08	1.38	16.90	0.02	cellulose degradation	Ulrich et al. (2008)
*Streptomyces*	0.15	0.02	0.15	0.24	cellulose degradation	Gong et al. (2020)
					hemicellulose degradation	Gu et al. (2012)
*Lachnospiraceae_NK4A136_group*	0.15	0.01	0.03	0.41	cellulose degradation	Zou et al. (2021)
*Paenibacillus*	0.01	0.06	10.05	0.31	cellulose degradation	Eida et al. (2012)
					pectin degradation	Ishihara et al. (2021)
*Acinetobacter*	1.21	42.09	34.72	0.03	cellulose degradation	Zhao et al. (2016)
					pectin degradation	Xue et al. (2009)
					Lignin degradation	Zhang et al. (2020a)
*Novosphingobium*	0.01	0.30	36.28	0.26	Lignin degradation	
*Comamonas*	0.05	0.01	53.83	0.10	Lignin degradation	
*Gordonia*	0.05	0.01	0.14	0.43	cellulose degradation	Woo et al. (2014)
*Pseudomonas*	0.28	4.09	14.48	0.05	cellulose degradation	Xu et al. (2018); Yu et al. (2021); Zhang et al. (2022)
					Lignin degradation	Yu et al. (2021)
					pectin degradation	
*Sphingobacterium*	0.23	3.76	16.09	0.06	cellulose degradation	Zhang et al. (2020b)

### 3.2 Isolation and identification of cellulose degrading bacteria of *G. cantor* larvae

#### 3.2.1 Isolation and screening of cellulose degrading bacteria

**FIGURE 5:**
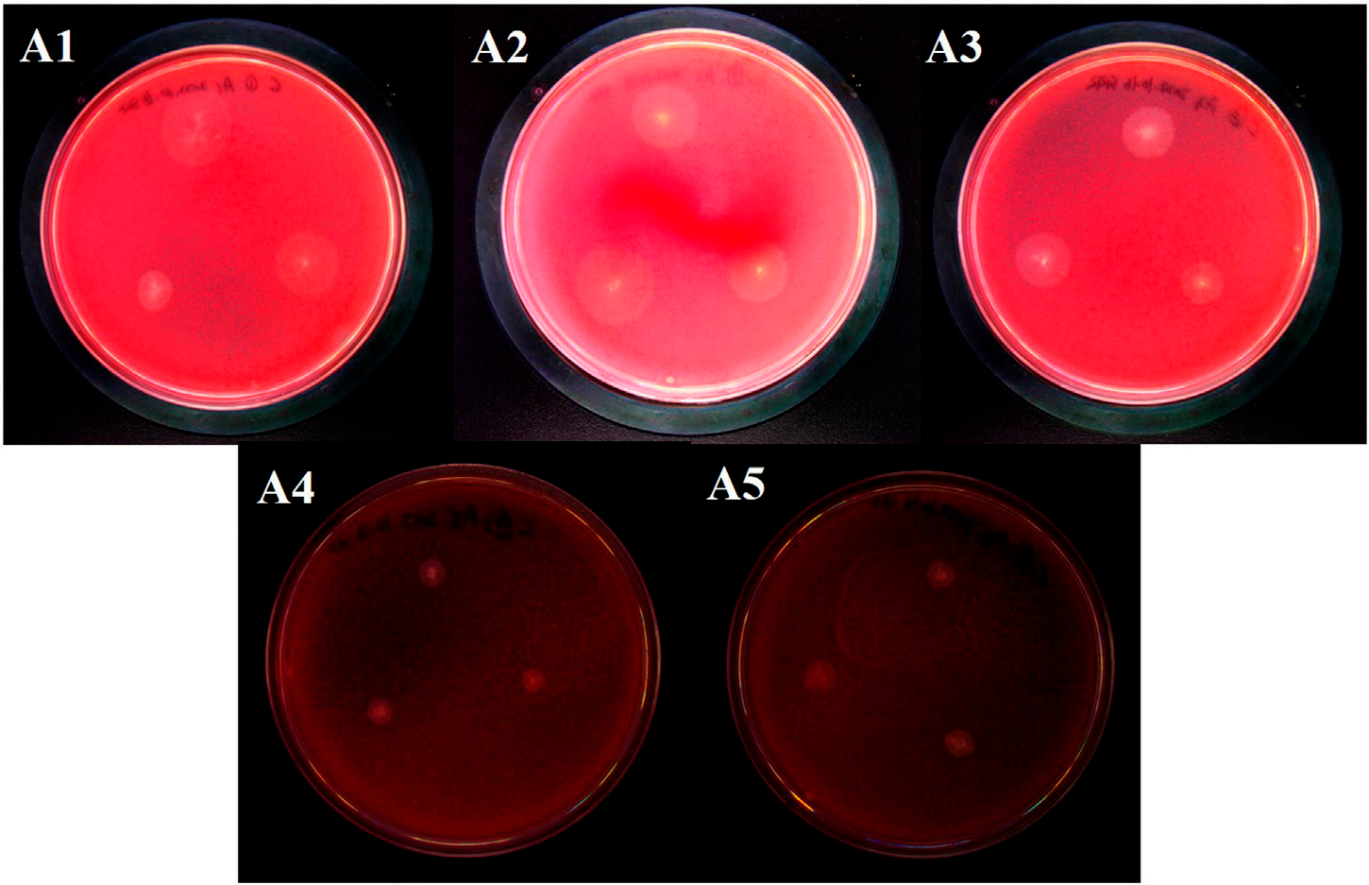
Cellulose-degrading bacteria derived from the intestinal tract of *G. cantor*.

The intestinal microorganisms of the *G. cantor* fourth-instar larvae were screened and cultivated for cellulose, and 5 strains of cellulose-degrading bacteria were isolated, named A1, A2, A3, A4 and A5 respectively (Figure 5). Each colony degraded cellulose and produced a transparent circle, colony diameter and transparent circle diameter were shown in the Table 3. A3 has the strongest ability to degrade cellulose, significantly higher than A1, A4 and A5, but not significantly different from A2, and A5 has the weakest ability to degrade cellulose.

**TABLE 3:** Colony diameters and cellulose degradation circles diameters of cellulose degrading bacteria derived from intestinal tract of *G. cantor*.

Strain number	Colony diameter d (mm)	Diameter of transparent ring D (mm)	Diameter of transparent ring D/Colony diameter d
A1	5.34 ± 0.59 a	11.94 ± 1.45 ab	2.22 ± 0.06 bc
A2	5.60 ± 0.38 a	14.74 ± 1.38 a	2.58 ± 0.12 ab
A3	4.74 ± 0.18 ab	13.68 ± 0.71 a	2.91 ± 0.13 a
A4	3.70 ± 0.20 b	8.98 ± 0.68 bc	2.41 ± 0.08 b
A5	3.87 ± 0.13 b	7.32 ± 0.17 c	1.91 ± 0.05 c

Note: The data in the table is average plus standard error. The diameter of each colony and D/d (D: the diameter of the transparent circle; d: the diameter of the colony) exhibited non-normal distribution. One-way ANOVA was performed after Lg transformation, and Tukey’s HSD was employed for multiple comparisons (*p* < 0.05). The diameter of the cellulose degradation circles also showed non-normal distribution, thus non-parametric tests were used for multiple comparisons. Different lowercase letters after each row of data indicated significant differences in enzyme activity between different strains.

The cellulase activity (CMCA) and filter paper enzyme activity (FPA) were measured using the DNSA method (Table 4). Among the 5 cellulose degrading bacteria strains, A3 and A4 had the highest cellulase activity measuring 94.09 ± 1.10 U/mL and 94.42 ± 0.42 U/mL, respectively. And the statistical analysis showed no significant difference compared to A1 and A2, while A5 had the lowest cellulase activity. The filter paper enzyme activity of A5 was the highest (127.46 ± 3.54 U/mL), but there was no significant difference compared to A1, A2 and A3. The filter paper enzyme activity of A4 was the lowest, and there was no difference between the A1, A2 and A3.

**TABLE 4:** Cellulase activity and filter paper activity of cellulose-degrading bacteria strains of *G. cantor*.

Strain number	CMCA (U/mL)	FPA (U/mL)
A1	86.43 ± 0.41 b	104.43 ± 0.24 b
A2	86.80 ± 1.24 b	102.78 ± 1.48 bc
A3	94.09 ± 1.10 a	99.00 ± 1.56 bc
A4	94.42 ± 0.42 a	95.25 ± 1.06 c
A5	73.49 ± 1.29 c	127.46 ± 3.54 a

Note: The data were average plus or minus standard error in the table. The cellulase activity and filter paper enzyme activity of the cellulose-degrading bacteria demonstrated normal distribution. ANOVA was used for one-way analysis of variance, and Tukey’s HSD was applied for multiple comparisons (*p* < 0.05). Different lowercase letters after each row of data indicated significant differences in enzyme activity between different strains.

#### 3.2.2 Identification of 5 strains cellulose-degrading bacteria

**FIGURE 6:**
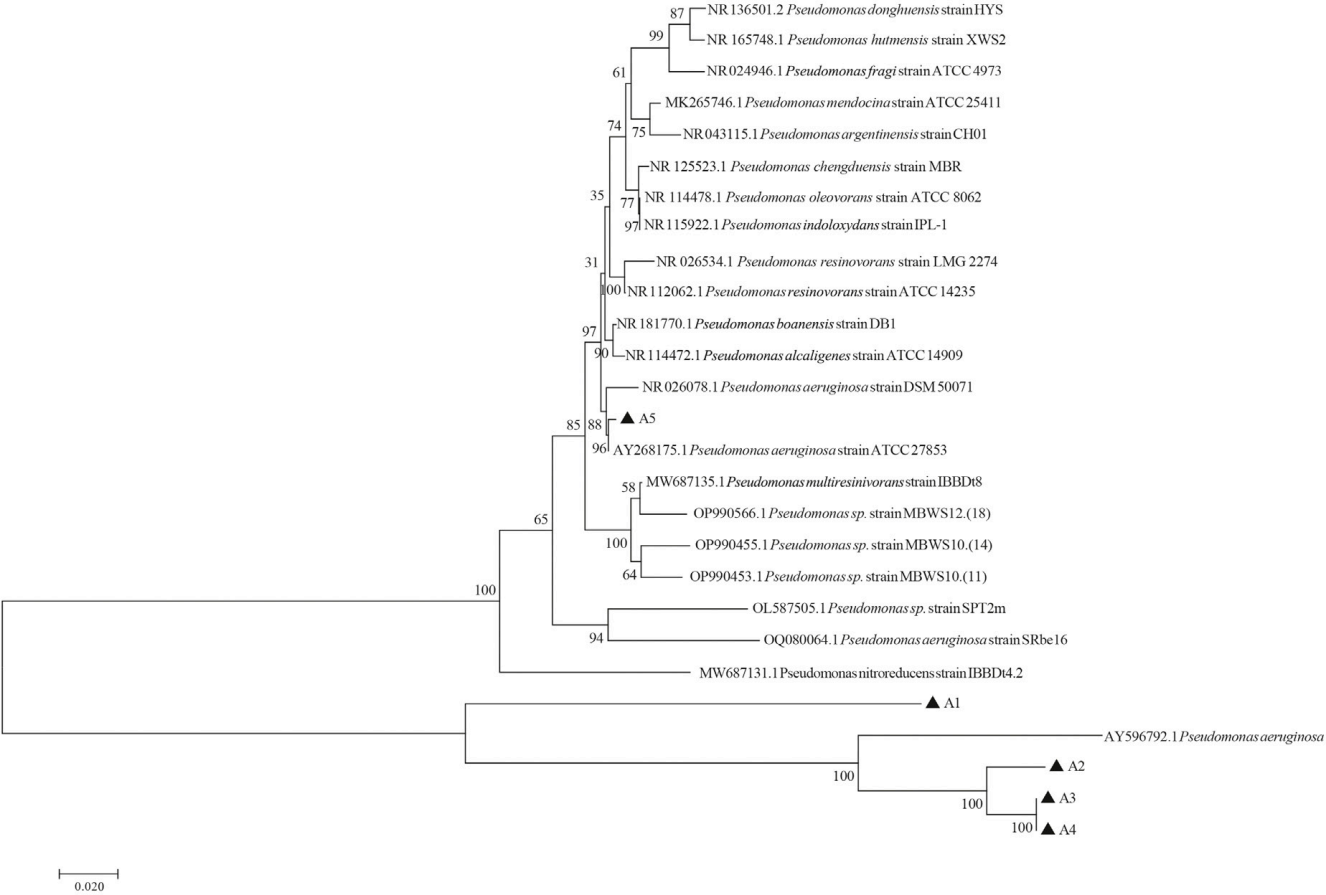
Phylogenetic tree of cellulose-degrading bacteria derived from the intestinal tract of *G. cantor* larvae constructed based on 16S rDNA.

All 5 cellulose degrading bacterial colonies were circular, white, opaque with protrusions, moist surfaces, and intact edges. No differences were observed in terms of colony morphology. Gram staining was performed on them separately, and the results were all pink (Supplementary Figure S2), indicating that they were Gram negative bacteria. Under the microscope, the strains appeared rod-shaped. DNA of five cellulose-degrading bacteria were extracted and used as a template for PCR amplification with 27F and 1492R as primers. The agarose gel electrophoresis results were shown in Supplementary Figure S3. The PCR product bands were about 1500 bp (Supplementary Data Sheet S2). By comparing the 16S rDNA sequence of five strains with that of NCBI strain, 1000 bootstraps were applied to the phylogenetic tree, it was found that five cellulose-degrading bacteria belonging to *Pseudomonas* (Figure 6). Based on the morphology and Gram staining results, 5 strains of cellulose-degrading bacteria were identified as *Pseudomonas aeruginosa*.

## 4 Discussion

The gut of *G. cantor* larvae exhibited a diverse and complex bacterial community and Proteobacteria was the most dominant phylum (Figure 3A). This phylum is widely recognized as the most common in insect gut bacteria, and similar findings have been reported in other studies. The dominant intestinal flora of *M. alternatus* and *A. chinensis* larvae also consisted of Proteobacteria (Rizzi et al., 2013; Hu et al., 2017). Similarly, the dominant bacterial phylum in *A. glabripennis* larvae was Proteobacteria, and it remained unaffected by the host plant (Scully et al., 2014). This suggests that different beetles may share common dominant bacteria. We hypothesize that certain genera, such as cellulose-degrading bacteria, may be associated with cellulose-degrading similarities observed in wood-eating insects like Proteobacteria. For instance, the cellulose-degrading bacteria *Ochrobactrum*, found in the gut of *B. horsfieldi* larvae and *Spodoptera frugiperda* larvae, both belong to the Proteobacteria phylum (Li et al., 2020b; Yang et al., 2021). The dominant bacteria genera were different between the larval gut and larval frass (Figure 3C), indicating that bacterial structure of *G. cantor* changed after food digestion in gut. Additionally, *Lactococcus* and *Enterococcus* were the two most abundant of larval gut samples. Some cellulolytic *Enterococcus* strains were found in the eri silkworm larvae, *Samia ricini* (MsangoSoko et al., 2021; Unban et al., 2022), since Enterobacteriales have been observed to assist in the breakdown of plant cell wall compounds, including pectin (Shil et al., 2014; Blankenchip et al., 2018). Therefore, *Enterococcus* may have more important role in cellulose degradation in *G. cantor* (Robert and Bernalier-Donadille, 2003).

The function of bacterial flora was mainly involved in carbohydrate metabolism and amino acid metabolism, partial lipid, nucleotide metabolism and biodegradation in the host (Figure 4B). Hence, it was speculated that apart from endogenous cellulase genes (Su et al., 2021), intestinal bacteria also play a significant role in the kapok-degraded cellulose feeding of beetles. In carbohydrate metabolism, the bacterial flora primarily participated in starch and sucrose metabolism, amino sugar and nucleotide sugar metabolism, and pyruvate metabolism (Figure 4C). The results indicated that the intestinal flora of beetle larvae plays a significant role in carbohydrate metabolism, so it was speculated that beetle gut system may modulate the composition and function of the flora to serve nutrient metabolic needs and aid in food digestion. Furthermore, insects can be capable of regulating their own intestinal microorganisms and acquiring beneficial bacteria to facilitate food digestion (Mira and Moran, 2002).

The functional prediction results revealed bacteria with cellulose-degrading abilities, which serve as a basis for subsequent screening of cellulose-degrading bacteria. 12 genera of bacteria reported cellulose degrading bacteria were found in *G. cantor* (Table 2), which is significant to their intestinal digestion mechanism. Based on the functional prediction results of isolation and identification experiments, five strains of cellulose-degrading bacteria were obtained (Figure 5). In particular, the abundance information ratio of cellulose-degrading bacteria, belonging to 4 genera of *Microbacterium*, *Streptomyces*, *Lachnospiraceae_NK4A136_group*, and *Gordonia,* were higher in the intestinal tract compared to fecal samples (Table 2). *Microbacterium* had been found in the intestines of other insects, such as *Stromatium barbatum* (Fabr.) and *Zootermopsis angusticollis* (Wenzel et al., 2002; Yadav et al., 2022). However, the successful isolation and culture of *Microbacterium*, *Streptomyces*, *Lachnospiraceae_NK4A136_group*, and *Gordonia* were derived from the external environment, such as soil (Soares et al., 2012; Woo et al., 2014; Gong et al., 2020; Zou et al., 2021), which were relatively rare from the gut. The results indicated that the cellulose-degrading bacteria in *G. cantor* which belonging to *Microbacterium*, *Streptomyces*, *Lachnospiraceae_NK4A136_group*, and *Gordonia* of the gut may not be easily cultured in the environment. Five strains of culturable cellulose-degrading bacteria were obtained from the intestinal tract of longhorn beetles by screening cellulose-derived bacteria (Figure 5). Combining their morphological and physiological characteristics, the strains were identified as *P*. *aeruginosa* (Figure 6), which belongs to the genus *Pseudomonas*. This finding was consistent with the predicted function of cellulose-degrading bacteria in the results of intestinal bacterial diversity analysis. Other common cellulose-degrading bacteria include *Bacillus* (Li et al., 2020b; Kumawat et al., 2021; Zhang et al., 2021), *Klebsiella* (Dar et al., 2021), *Enterococcus* (MsangoSoko et al., 2021), and *Pseudomonas* sp. (Zhang et al., 2022).

The laccase, produced by *Pseudomonas*, had the capability to disintegrate the crystal structure of cellulose, unblock lignin on cellulose and hemicellulose, and enhance the ability to degrade cellulose (Zhang et al., 2007). *P*. *aeruginosa* was capable of producing alkaline cellulase. Previous studies had revealed that the main function of this enzyme was to break down the crystalline structure of cellulose, facilitate the release of amorphous cellulose and enhance the saccharification of fiber in an alkaline environment. Additionally, a portion of the cellulose was converted into simple sugars (Lu et al., 2017). *Pseudomonas* sp. may play a role in the degradation of secondary metabolites in host plant, which were associated with defense substances specific to the host plant. For instance, *P*. *aeruginosa* had demonstrated the capability to degrade linalool, which was isolated from *Pagiophloeus tsushimanus* (Qiao et al., 2023). Additionally, *Pseudomonas* sp. was obtained from the gut of mountain pine beetle *Dendroctonus ponderosae* and had shown the ability to degrade terpenes (Adams et al., 2013). *Pseudomonas* sp. had exhibited the capacity to degrade α-pinene and displayed resistance to high levels of α-pinene which isolated from the gut of the red turpentine beetle *D*. *valens* LeConte (Scolytinae) (Xu et al., 2016). 3-hexanone, decanal, nonanal and para-xylene (p-xylene) had an attractive effect on both male and female adults of *G. cantor*, which were compounds from the kapok (Dong, 2021). Additionally, *Pseudomonas* could somehow support the monophagous feeding habit of *Brassicogethes matronalis* (Teoh et al., 2021). It is hypothesized that this kapok consuming activity may be also attributed to intestinal bacteria in *G. cantor*. Further research is needed to investigate whether the *P. aeruginosa* from *G. cantor* plays a role in the degradation of these secondary metabolites.

The screening of cellulose-degrading bacteria yielded only 5 target strains in the intestinal tract of *G. cantor*. This number was significantly lower compared to the 12 genera of cellulose-degrading bacteria identified in the OTUs annotation results of 16S rDNA sequencing. These findings suggest that obtaining the desired target strain of cellulose-degrading bacteria from the intestinal source may be challenging due to various factors such as temperature, aerobic/anaerobic conditions, pH value, special nutrients, and microbial interactions in the environment. However, it is important to acknowledge that the study has certain limitations.

## Data Availability

The datasets presented in this study can be found in online repositories. The names of the repository/repositories and accession number(s) can be found in the article/Supplementary Material.
