# Supplementary material for: Characterization of bacterial diversity and screening of cellulose-degrading bacteria in the gut system of Glenea cantor (Fabricius) larvae
Source: Front Bioeng Biotechnol. 2024 Feb 22;12:1340168. doi: 10.3389/fbioe.2024.1340168 (PMC10919226; doi:10.3389/fbioe.2024.1340168)
Supplement: Supplementary file 1 [file Table1.docx]

## Supplementary materials

**Table S1 Information table of the fourth instar larvae of *Glenea cantor***

| **Items** | **Weight (g)** | **Length (cm)** | **Intestinal length (cm)** | **Intestinal weight (g)** | **Frass weight (g）** |
| --- | --- | --- | --- | --- | --- |
| Biological duplication 1 | 0.234 ± 0.026 | 2.46 ± 0.14 | 4.88 ± 0.82 | 0.252 | 17.683 |
| Biological duplication 2 | 0.229 ± 0.030 | 2.58 ± 0.18 | 4.42 ± 0.94 | 0.213 | 20.413 |
| Biological duplication 3 | 0.326 ± 0.059 | 2.91 ± 0.19 | 5.04 ± 0.77 | 0.269 | 16.291 |

Note: Each group contained 5 larvae. Starvation duration: December 14 to December 16, 2019; Frass collection duration: November 28 to December 4, 2019.
