# Supplementary figures and images for: Characterization of bacterial diversity and screening of cellulose-degrading bacteria in the gut system of Glenea cantor (Fabricius) larvae
Source: Front Bioeng Biotechnol. 2024 Feb 22;12:1340168. doi: 10.3389/fbioe.2024.1340168 (PMC10919226; doi:10.3389/fbioe.2024.1340168)

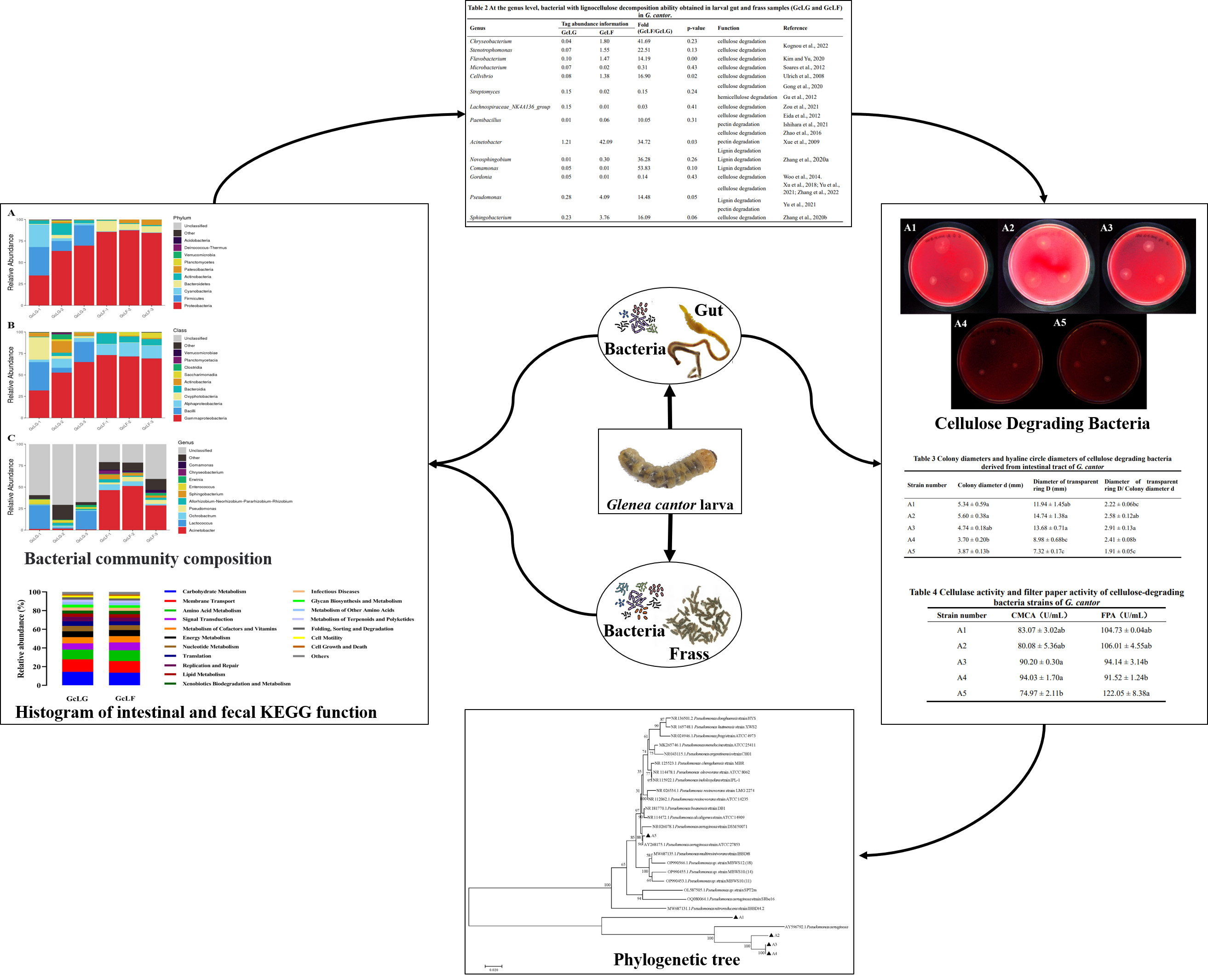

Supplement: Supplementary file 2 [file Image1.JPEG]
