# Supplementary material for: Characterization of bacterial diversity and screening of cellulose-degrading bacteria in the gut system of Glenea cantor (Fabricius) larvae
Source: Front Bioeng Biotechnol. 2024 Feb 22;12:1340168. doi: 10.3389/fbioe.2024.1340168 (PMC10919226; doi:10.3389/fbioe.2024.1340168)
Supplement: Supplementary file 3 [file Table2.docx]

## Supplementary materials

**Table S2 Segmented pH of the digestive tract of 4-day-old larvae in *G. cantor***

| **Items** | **Foregut** | **Midgut-1** | **Midgut-2** | **Midgut-3** | **Midgut-4** | **Hindgut** |
| --- | --- | --- | --- | --- | --- | --- |
| Biological duplication 1 | 6.60 ± 0.10 | 5.77 ± 0.03 | 6.17 ± 0.03 | 7.97 ± 0.17 | 8.20 ± 0.12 | 6.83 ± 0.07 |
| Biological duplication 2 | 6.57 ± 0.12 | 5.87 ± 0.07 | 6.33 ± 0.03 | 7.93 ± 0.09 | 8.30 ± 0.15 | 6.43 ± 0.03 |
| Biological duplication 3 | 6.57 ± 0.12 | 6.00 ± 0.10 | 6.20 ± 0.06 | 8.07 ± 0.09 | 8.30 ± 0.26 | 6.63 ± 0.07 |
