# Supplementary material for: Characterization of bacterial diversity and screening of cellulose-degrading bacteria in the gut system of Glenea cantor (Fabricius) larvae
Source: Front Bioeng Biotechnol. 2024 Feb 22;12:1340168. doi: 10.3389/fbioe.2024.1340168 (PMC10919226; doi:10.3389/fbioe.2024.1340168)
Supplement: Supplementary file 4 [file Table3.docx]

## Supplementary materials

**Table S3 Main c****ulture medium used in the experiment**

| Medium | Component |
| --- | --- |
| Enrichment medium | 5 g sodium carboxymethyl cellulose, 5 g beef extract, 1 g peptone, 5 g fermented straw powder, 5 g NaCl, add water to make the volume to 1 L, and adjust the pH to 7.0. |
| Congo red cellulose sodium medium | 1.0 g sodium nitrate, 1.2 g disodium hydrogen phosphate, 0.9 g dipotassium hydrogen phosphate, 0.5 g magnesium sulfate, 0.5 g potassium chloride, 0.5 g yeast powder, 0.5 g acid hydrolyzed casein, 0.2 g Congo red, 5.0 g cellulose powder, 15 g agar powder, add water to make the volume to 1 L, and adjust the pH to 7.0. |
| LB solid medium | 15 g agar powder, 10 g sodium chloride, 10 g tryptone, 5 g yeast powder, add water to make the volume to 1 L, adjust the pH to 7.0, do not add agar powder to the liquid. |
| Carboxymethyl cellulose sodium medium | 1.5 g potassium dihydrogen phosphate, 2.5 g disodium hydrogen phosphate, 20.0 g sodium carboxymethylcellulose, 2.5 g peptone, 0.5 g yeast extract, 15 g agar powder, add water to make the volume to 1L, adjust the pH to 7.0, the liquid should not be Add agar powder. |
| Enzyme-producing medium | Peptone 10.0 g, beef extract 5.0 g, NaCl 5.0 g, sodium carboxymethyl cellulose 5 g, add water to make the volume to 1000 mL, and adjust the pH to 7.0. |
