# Supplementary material for: Characterization of bacterial diversity and screening of cellulose-degrading bacteria in the gut system of Glenea cantor (Fabricius) larvae
Source: Front Bioeng Biotechnol. 2024 Feb 22;12:1340168. doi: 10.3389/fbioe.2024.1340168 (PMC10919226; doi:10.3389/fbioe.2024.1340168)
Supplement: Supplementary file 5 [file DataSheet2.docx]

## Supplementary materials

The nucleotide sequence of 16S rDNA PCR amplification of cellulolytic bacteria from intestinal tract of *G. cantor* were list below.

>A1

TGAGGTGCGAAAGCGTGGGGAGAAACAGGATTAGATACCCTGGTAGTCCACGCCGTAAACGATGTCGACTAGCCGTTGGGATCCTTGAGATCTTAGTGGCGCAGCTAACGCGATAAGTCGACCGCCTGGGGAGTACGGCCGCAAGGTTAAAACTCAAATGAATTGACGGGGGCCCGCACAAGCGGTGGAGCATGTGGTTTAATTCGAAGCAACGCGAAGAACCTTACCTGGCCTTGACATGCTGAGAACTTTCCAGAGATGGATTGGTGCCTTCGGGAACTCAGACACAGGTGCTGCATGGCTGTCGTCAGCTCGTGTCGTGAGATGTTGGGTTAAGTCCCGTAACGAGCGCAACCCTTGTCCTTAGTTACCAGCACCTCGGGTGGGCACTCTAAGGAGACTGCCGGTGACAAACCGGAGGAAGGTGGGGATGACGTCAAGTCATCATGGCCCTTACGGCCAGGGCTACACACGTGCTACAATGGTCGGTACAAAGGGTTGCCAAGCCGCGAGGTGGAGCTAATCCCATAAAACCGATCGTAGTCCGGATCGCAGTCTGCAACTCGACTGCGTGAAGTCGGAATCGCTAGTAATCGTGAATCAGAATGTCACGGTGAATACGTTCCCGGGCCTTGTACACACCGCCCGTCACACCATGGGAGTGGGTTGCTCCAGAAGTAGCTAGTCTAACCGCAAGGGGGACGGTTACCACGGAGTGATTCATGACTGGGGTGAAGTCGTAACAAGGGTAGCACTCATCGTTTACGGCGTGGACTACCAGGGTATCTAATCCTGTTTGCTCCCCACGCTTTCGCCTCAGTGTCAGTATCAGTCCAGGTGGTCGCCTTCGCCACTGGTGTTCCTTCCTATATCTACGCATTTCACCGCTACACAGGAAATTCCACCACCCTCTACCGTACTCTAGCTCAGTAGTTTTGGATGCAGTTCCCAGGTTGAGCCCGGGGATTTCACATCCAACTTGCTGAACCACCTACGCGCGCTTTACGCCCAGTAATTCCGATTAACGCTTGCACCCTTCGTATTACCGCGGCTGCTGGCACGAAGTTAGCCGGTGCTTATTCTGTTGGTAACGTCAAAACAGCAAGGTATTAACTTACTGCCCTTCCTCCCAACTTAAAGTGCTTTACAATCCGAAGACCTTCTTCACACACGCGGCATGGCTGGATCAGGCTTTCGCCCATTGTCCAATATTCCCCACTGCTGCCTCCCGTAGGAGTCTGGACCGTGTCTCAGTTCCAGTGTGACTGATCATCCTCTCAGACCAGTTACGGATCGTCGCCTTGGTAGGCCTTTACCCCACCAACTAGCTAATCCGACCTAGGCTCATCTGATAGCGTGAGGTCCGAAGATCCCCCACTTTCTCCCTCAGGACGTATGCGGTATTAGCGCCCGTTTCCGGACGTTATCCCCCACTACCAGGCAGATTCCTAGGCATTACTCACCCGTCCGCCGCTGAATCCGGAAGCAAGCTCCCTTCATCCGCTCGACTTGCATGTGTTAGGCCTGCCGCCAGCGTTCAATCTGAGCAG

>A2

CAGTCATGAATCACTCCGTGGTAACCGTCCCCCTTGCGGTTAGACTAGCTACTTCTGGAGCAACCCACTCCCATGGTGTGACGGGCGGTGTGTACAAGGCCCGGGAACGTATTCACCGTGACATTCTGATTCACGATTACTAGCGATTCCGACTTCACGCAGTCGAGTTGCAGACTGCGATCCGGACTACGATCGGTTTTATGGGATTAGCTCCACCTCGCGGCTTGGCAACCCTTTGTACCGACCATTGTAGCACGTGTGTAGCCCTGGCCGTAAGGGCCATGATGACTTGACGTCATCCCCACCTTCCTCCGGTTTGTCACCGGCAGTCTCCTTAGAGTGCCCACCCGAGGTGCTGGTAACTAAGGACAAGGGTTGCGCTCGTTACGGGACTTAACCCAACATCTCACGACACGAGCTGACGACAGCCATGCAGCACCTGTATCAGCAGTGGCGAAGGCACCAATCCATCTCTGGAAAGTTCTCAGCATGTCAAGGCCAGGTAAGGTTCTTCGCGTTGCTTCGAATTAAACCACATGCTCCACCGCTTGTGCGGGCCCCCGTCAATTCATTTGAGTTTTAGTCTTGCGACCGTACTCCCCAGGCGGAGTGCTTAATGCGTTAGCTGCAGCACTAAAGGGCGGAAACCCTCTAACACTTAGCACTCATCGTTTACGGCGTGGACTACCAGGGTATCTAATCCTGTTTGCTCCCCACGCTTTCGCGCCTCAGTGTCAGTATCAGTCCAGGTGGTCGCCTTCGCCACTGGTGTTCCTTCCTATATCTACGCATTTCACCGCTACACAGGAAATTCCACCACCCTCTACCGTACTCTAGCTCAGTAGTTTTGGATGCAGTTCCCAGGTTGAGCCCGGGGATTTCACATCCAACTTGCTGAACCACCTACGCGCGCTTTACGCCCAGTAATTCCGATTAACGCTTGCACCCTTCGTATTACCGCGGCTGCTGGCACGAAGTTAGCCGGTGCTTATTCTGTTGGTAACGTCAAAACAGCAAGGTATTAACTTACTGCCCTTCCTCCCAACTTAAAGTGCTTTACAATCCGAAGACCTTCTTCACACACGCGGCATGGCTGGATCAGGCTTTCGCCCATTGTCCAATATTCCCCACTGCTGCCTCCCGTAGGAGTCTGGACCGTGTCTCAGTTCCAGTGTGACTGATCATCCTCTCAGACCAGTTACGGATCGTCGCCTTGGTAGGCCTTTACCCCACCAACTAGCTAATCCGACCTAGGCTCATCTGATAGCGTGAGGTCCGAAGATCCCCCACTTTCTCCCTCAGGACGTATGCGGTATTAGCGCCCGTTTCCGGACGTTATCCCCCACTACCAGGCAGATTCCTAGGCATTACTCACCCGTCCGCCGCTGAATCCGGAAGCAAGCTCCCTTCATCCGCTCGACTTGCATGTGTTAGGCCTGCCGCCAGCGTTCAATCTGAGCCAGATCCAAACTCTA

>A3

CCGTCCCCCTTGCGGTTAGACTAGCTACTTCTGGAGCAACCCACTCCCATGGTGTGACGGGCGGTGTGTACAAGGCCCGGGAACGTATTCACCGTGACATTCTGATTCACGATTACTAGCGATTCCGACTTCACGCAGTCGAGTTGCAGACTGCGATCCGGACTACGATCGGTTTTATGGGATTAGCTCCACCTCGCGGCTTGGCAACCCTTTGTACCGACCATTGTAGCACGTGTGTAGCCCTGGCCGTAAGGGCCATGATGACTTGACGTCATCCCCACCTTCCTCCGGTTTGTCACCGGCAGTCTCCTTAGAGTGCCCACCCGAGGTGCTGGTAACTAAGGACAAGGGTTGCGCTCGTTACGGGACTTAACCCAACATCTCACGACACGAGCTGACGACAGCCATGCAGCACCTGTGTCTGAGTTCCCGAAGGCACCAATCCATCTCTGGAAAGTTCTCAGCATGTCAAGGCCAGGTAAGGTTCTTCGCGTTGCTTCGAATTAAACCACATGCTCCACCGCTTGTGCGGGCCCCCGTCAATTCATTTGAGTTTTAACCTTGCGGCCGTACTCCCCAGGCGGTCGACTTATCGCGTTAGCTGCGCCACTAAGATCTCAAGGATCCCAACGGCTAGTCGACATCGTTTACGGCGTGGACTACCAGGGTATCTAATCCTGTTTGCTCCCCACGCTTTCGCACCTCAGTGTCAGTATCAGTCCAGGTGGTCGCCTTCGCCACTGGTGTTCCTTCCTATATCTACGCATTTCACCGCTACACAGGAAATTCCACCACCCTCTACCGTACTCTAGCTCAGTAGTTTTGGATGCAGTTCCCAGGTTGAGCCCGGGGATTTCACATCCAACTTGCTGAACCACCTACGCGCGCTTTACGCCCAGTAATTCCGATTAACGCTTGCACCCTTCGTATTACCGCGGCTGCTGGCACGAAGTTAGCCGGTGCTTATTCTGTTGGTAACGTCAAAACAGCAAGGTATTAACTTACTGCCCTTCCTCCCAACTTAAAGTGCTTTACAATCCGAAGACCTTCTTCACACACGCGGCATGGCTGGATCAGGCTTTCGCCCATTGTCCAATATTCCCCACTGCTGCCTCCCGTAGGAGTCTGGACCGTGTCTCAGTTCCAGTGTGACTGATCATCCTCTCAGACCAGTTACGGATCGTCGCCTTGGTAGGCCTTTACCCCACCAACTAGCTAATCCGACCTAGGCTCATCTGATAGCGTGAGGTCCGAAGATCCCCCACTTTCTCCCTCAGGACGTATGCGGTATTAGCGCCCGTTTCCGGACGTTATCCCCCACTACCAGGCAGATTCCTAGGCATTACTCACCCGTCCGCCGCTGAATCCAGGAGCAAGCTCCCTTCATCCGCTCGACTG

>A4

CCTGTTACGACTTCACCCCAGTCATGAATCACTCCGTGGTAACCGTCCCCCTTGCGGTTAGACTAGCTACTTCTGGAGCAACCCACTCCCATGGTGTGACGGGCGGTGTGTACAAGGCCCGGGAACGTATTCACCGTGACATTCTGATTCACGATTACTAGCGATTCCGACTTCACGCAGTCGAGTTGCAGACTGCGATCCGGACTACGATCGGTTTTATGGGATTAGCTCCACCTCGCGGCTTGGCAACCCTTTGTACCGACCATTGTAGCACGTGTGTAGCCCTGGCCGTAAGGGCCATGATGACTTGACGTCATCCCCACCTTCCTCCGGTTTGTCACCGGCAGTCTCCTTAGAGTGCCCACCCGAGGTGCTGGTAACTAAGGACAAGGGTTGCGCTCGTTACGGGACTTAACCCAACATCTCACGACACGAGCTGACGACAGCCATGCAGCACCTGTGTCTGAGTTCCCGAAGGCACCAATCCATCTCTGGAAAGTTCTCAGCATGTCAAGGCCAGGTAAGGTTCTTCGCGTTGCTTCGAATTAAACCACATGCTCCACCGCTTGTGCGGGCCCCCGTCAATTCATTTGAGTTTTAACCTTGCGGCCGTACTCCCCAGGCGGTCGACTTATCGCGTTAGCTGCGCCACTAAGATCTCAAGGATCCCAACGGCTAGTCGACATCGTTTACGGCGTGGACTACCAGGGTATCTAATCCTGTTTGCTCCCCACGCTTTCGCACCTCAGTGTCAGTATCAGTCCAGGTGGTCGCCTTCGCCACTGGTGTTCCTTCCTATATCTACGCATTTCACCGCTACACAGGAAATTCCACCACCCTCTACCGTACTCTAGCTCAGTAGTTTTGGATGCAGTTCCCAGGTTGAGCCCGGGGATTTCACATCCAACTTGCTGAACCACCTACGCGCGCTTTACGCCCAGTAATTCCGATTAACGCTTGCACCCTTCGTATTACCGCGGCTGCTGGCACGAAGTTAGCCGGTGCTTATTCTGTTGGTAACGTCAAAACAGCAAGGTATTAACTTACTGCCCTTCCTCCCAACTTAAAGTGCTTTACAATCCGAAGACCTTCTTCACACACGCGGCATGGCTGGATCAGGCTTTCGCCCATTGTCCAATATTCCCCACTGCTGCCTCCCGTAGGAGTCTGGACCGTGTCTCAGTTCCAGTGTGACTGATCATCCTCTCAGACCAGTTACGGATCGTCGCCTTGGTAGGCCTTTACCCCACCAACTAGCTAATCCGACCTAGGCTCATCTGATAGCGTGAGGTCCGAAGATCCCCCACTTTCTCCCTCAGGACGTATGCGGTATTAGCGCCCGTTTCCGGACGTTATCCCCCACTACCAGGCAGATTCCTAGGCATTACTCACCCGTCCGCCGCTGAATCCGGAAGCAAGCTCCCTTCATCCGCTCGACTTGCATGTGTTAGGCCTGCCGCCAGCGTTCAATCTGAGCCAGGATCCAAACTC

>A5

TTAGAGTTTGGATCCTGGCTCAGATTGAACGCTGGCGGCAGGCCTAACACATGCAAGTCGAGCGGATGAAGGGAGCTTGCTTCCGGATTCAGCGGCGGACGGGTGAGTAATGCCTAGGAATCTGCCTGGTAGTGGGGGATAACGTCCGGAAACGGGCGCTAATACCGCATACGTCCTGAGGGAGAAAGTGGGGGATCTTCGGACCTCACGCTATCAGATGAGCCTAGGTCGGATTAGCTAGTTGGTGGGGTAAAGGCCTACCAAGGCGACGATCCGTAACTGGTCTGAGAGGATGATCAGTCACACTGGAACTGAGACACGGTCCAGACTCCTACGGGAGGCAGCAGTGGGGAATATTGGACAATGGGCGAAAGCCTGATCCAGCCATGCCGCGTGTGTGAAGAAGGTCTTCGGATTGTAAAGCACTTTAAGTTGGGAGGAAGGGCAGTAAGTTAATACCTTGCTGTTTTGACGTTACCAACAGAATAAGCACCGGCTAACTTCGTGCCAGCAGCCGCGGTAATACGAAGGGTGCAAGCGTTAATCGGAATTACTGGGCGTAAAGCGCGCGTAGGTGGTTCAGCAAGTTGGATGTGAAATCCCCGGGCTCAACCTGGGAACTGCATCCAAAACTACTGAGCTAGAGTACGGTAGAGGGTGGTGGAATTTCCTGTGTAGCGGTGAAATGCGTAGATATAGGAAGGAACACCAGTGGCGAAGGCGACCACCTGGACTGATACTGACACTGAGGTGCGAAAGCGTGGGGAGCAAACAGGATTAGATACCCTGGTAGTCCACGCCGTAAACGATGTCGGCTAGCCGTTGGGATCCTTGAGATCTTAGTGGCGCAGCTAACGCGATAAAGTCGACCGCCTGGGGAGTACGGCCGCAAGGTTAAAACTCAAATGAATTGACGGGGGCCCGCACAAGCGGTGGAGCATGTGGTTTAATTCGAAGCAACGCGAAGAACCTTACCTGGCCTTGACATGCTGAGAACTTTCCAGAGATGGATTGGTGCCTTCGCCAACTCAGATACAGGTGCTGCATGGCTGTCGTCAGCTCGTGTCGTGAGATGTTGGGTTAAGTCCCGTAACGAGCGCAACCCTTGTCCTTAGTTACCAGCACCTCGGGTGGGCACTCTAAGGAGACTGCCGGTGACAAACCGGAGGAAGGTGGGGATGACGTCAAGTCATCATGGCCCTTACGGCCAGGGCTACACACGTGCTACAATGGTCGGTACAAAGGGTTGCCAAGCCGCGAGGTGGAGCTAATCCCATAAAACCGATCGTAGTCCGGATCGCAGTCTGCAACTCGACTGCGTGAAGTCGGAATCGCTAGTAATCGTGAATCAGAATGTCACGGTGAATACGTTCCCGGGCCTTGTACACACCGCCCGTCACACCATGGGAGTGGGTTGCTCCAGAAGTAGCTAGTCTAACCGCAAGGGGACGGTTACCACGGAGTGATTCATGACTGGGGTGAAGTCGTAACAAGGTAGCCG
