# Supplementary material for: Characterization of bacterial diversity and screening of cellulose-degrading bacteria in the gut system of Glenea cantor (Fabricius) larvae
Source: Front Bioeng Biotechnol. 2024 Feb 22;12:1340168. doi: 10.3389/fbioe.2024.1340168 (PMC10919226; doi:10.3389/fbioe.2024.1340168)
Supplement: Supplementary file 6 [file Table4.docx]

## Supplementary materials

**Preparation of Standard Curve.**

The standard curve preparation involved several steps. Firstly, 0.5 g of anhydrous glucose was weighed using an electronic balance. It was then dissolved in distilled water to make a 1 mg/mL glucose standard solution with a total volume of 500 mL. Next, 11 test tubes were used, numbered from 0 to 10. Sequentially, glucose standard solution was added to each tube in volumes of 0, 0.2, 0.4, 0.6, 0.8, 1.0, 1.2, 1.4, 1.6, 1.8, and 2.0 mL, respectively. Each tube was diluted to a final volume of 2 mL with distilled water (Table S4). After that, 2.5 mL of DNS reagent was added to each tube and mixed thoroughly. The tubes were then heated in a boiling water bath for 5 minutes. After cooling, the volume of the reaction solution in each tube was adjusted to 10 mL with distilled water and the absorbance of the solution was measured at a wavelength of 540 nm. Finally, a glucose standard curve was plotted using the glucose concentration as the x-axis and the absorbance value at 540 nm as the y-axis. The regression equation was derived from this curve.

**Table S4 Glucose standard curve reaction system**

| Number  Reagent | 0 | 1 | 2 | 3 | 4 | 5 | 6 | 7 | 8 | 9 | 10 |
| --- | --- | --- | --- | --- | --- | --- | --- | --- | --- | --- | --- |
| Distilled water (mL) | 2.0 | 1.8 | 1.6 | 1.4 | 1.2 | 1.0 | 0.8 | 0.6 | 0.4 | 0.2 | 0 |
| Glucose standard solution (mL) | 0 | 0.2 | 0.4 | 0.6 | 0.8 | 1.0 | 1.2 | 1.4 | 1.6 | 1.8 | 2.0 |
| Glucose content (mg) | 0 | 0.2 | 0.4 | 0.6 | 0.8 | 1.0 | 1.2 | 1.4 | 1.6 | 1.8 | 2.0 |


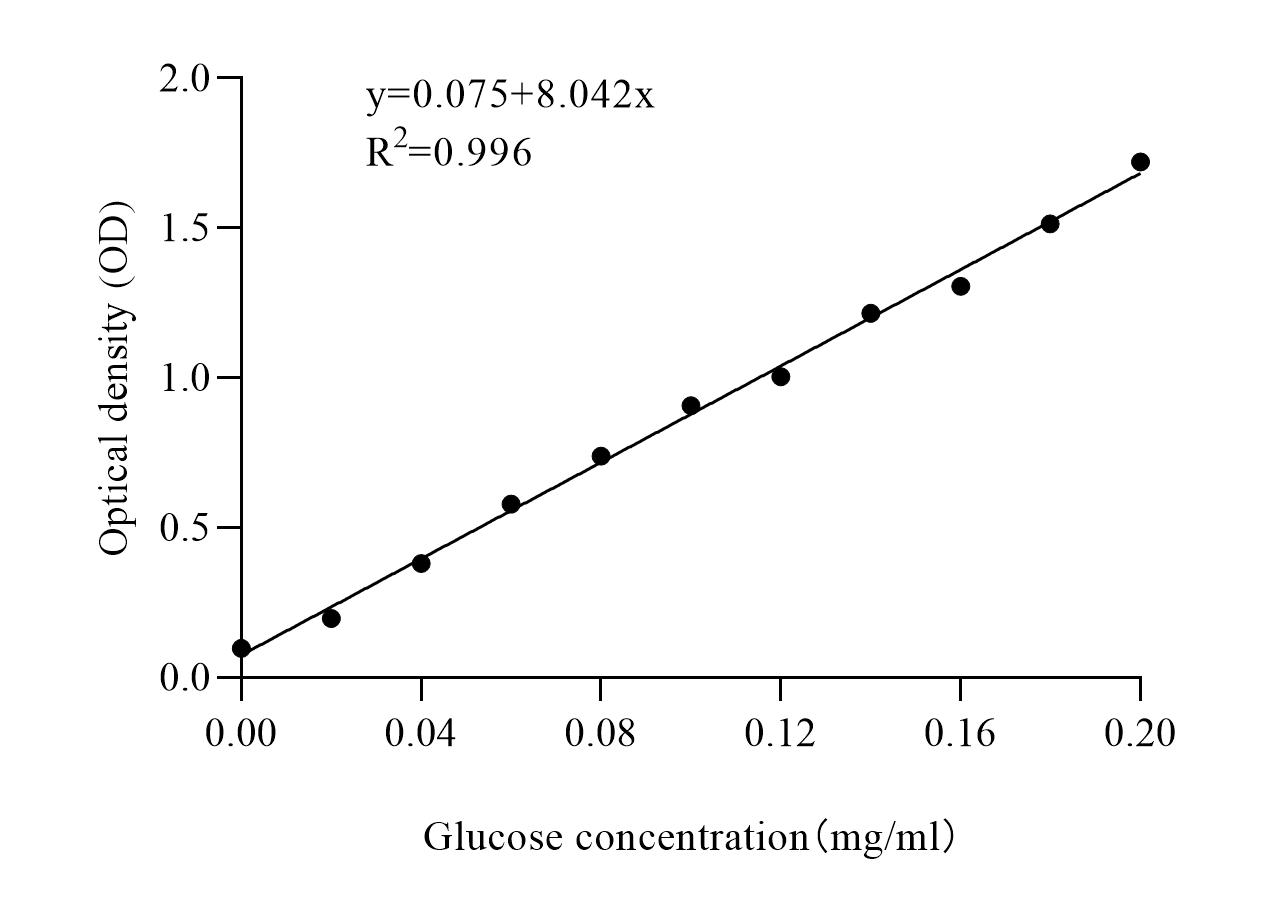


**Figure S1 Glucose standard curve**
