# Supplementary material for: Characterization of bacterial diversity and screening of cellulose-degrading bacteria in the gut system of Glenea cantor (Fabricius) larvae
Source: Front Bioeng Biotechnol. 2024 Feb 22;12:1340168. doi: 10.3389/fbioe.2024.1340168 (PMC10919226; doi:10.3389/fbioe.2024.1340168)
Supplement: Supplementary file 7 [file DataSheet1.docx]

## Supplementary materials

The raw data were submitted to the Sequence Read Archive (SRA) of NCBI under the accession number SRR13523916 - SRR13523921.
